# Public health implications of microbial food safety and foodborne diseases in developing countries

**DOI:** 10.3402/fnr.v60.29819

**Published:** 2016-11-08

**Authors:** Olumide A. Odeyemi

**Affiliations:** Springforth Scientific Resource Centre, Ile-Ife, Osun State, Nigeria

Food is one of the most important transmission routes of diseases globally due to microbial contaminations (1). Global emergence and re-emergence of foodborne pathogens have made microbiological safety and quality of food of public and health important (1, 2). Globally, more than 250 sources of foodborne diseases have been identified (3). Due to the increase of foodborne infectious diseases, several food quality regulations have been imposed in various countries. According to Grace, there is dearth of information regarding foodborne diseases in developing countries (4). Food contamination from microbial sources includes bacteria, protozoans, viruses, and fungi (5, 6). Consumption of food contaminated with foodborne pathogens and microbial by-products such as toxins could result in serious illnesses and economic loses (7). Currently, more than 2 million deaths occur every year in developing countries due to foodborne diseases, which are among more than 13 zoonoses implicated in over 2 billion illnesses worldwide (8). Those mostly affected are aged people, infants, children, and people with immunocompromised immune systems due to weakened immune system. It is therefore important that public health is taken into serious considerations in developing countries. In Africa, over 91 million people are affected according to recent report by the World Health Organization. It was also stated that 2.2 million children die of diarrhea every year in developing countries, while more than 600,000 children are reported to have died on yearly basis as a result of consumption of unsafe food in Southeast Asia (9). Among food implicated in foodborne diseases in developing countries are food from animal sources, fresh produce, and street-vended foods (4).

Over the years, safety and quality of food produced for human consumption in developing countries continue to increase because of foodborne disease outbreaks attributed to unsafe raw food, abused temperature, poor storage infrastructures, inadequate cooking, poor personal hygiene, improper handling methods, and cross- contamination of cooked food with uncooked raw food (1, 2, 10, 11). Food production in developing countries takes place mostly at home. Home serves as breeding ground for outbreak and spread of foodborne diseases. Personal hygiene of food handlers is, therefore, important to prevent outbreaks. In a recent study, it was observed that food handlers in Ghana, West Africa, lack knowledge of appropriate temperature for holding food and do not have adequate knowledge of sources of either contamination or cross-contamination (12). It was also observed in the study that water serves as most common route of transmission of foodborne pathogens. There are many factors influencing occurrence of foodborne diseases in developing countries, which when properly addressed can lead to reduction in occurrence of these diseases. Firstly, homes in developing countries serve as a key contributor to foodborne disease outbreaks due to the contamination of raw food with prepared food, lack of food safety awareness, poor personal hygiene, improper food handling, and preparation at home (13). Apart from contamination of food at home, other sources include farm, supply chain, consumers and food vendors, and lack of proper implementation of hazard analysis critical control point measures during food production (11). Some consumers store food at inappropriate temperatures, use contaminated cutting board, prepare food with unwashed hands, and store both raw and cooked food together, thereby causing cross-contamination (14, 15). It is, therefore, important that both food handlers and consumers are enlightened about the need for personal hygiene and food safety awareness as studies have shown that there is a strong correlation between food safety awareness and food safety attitude (12, 16, 17).

In developing countries, most foodborne disease outbreaks are underreported or underestimated. For example, Nigeria is a country with over 170 million people. However, it was reported that only 90,000 cases of foodborne diseases occur annually. Australia is a developed country with just 24 million people equivalent to 1:7 when compared to Nigeria. Yet more than 5.2 million people are reported to have foodborne diseases annually in Australia despite the high standard of living, good water supply, proactive government initiatives, and measures on food safety. It could be deduced from this fact that at least 36 million people (7×5.2 million) are possibly affected every year in Nigeria. Hence, underestimation of incidence of foodborne diseases in developing countries will affect the kind of measures and strategies implemented to curb foodborne disease outbreaks.

In conclusion, economic and public health implications of foodborne diseases in developing countries cannot be overestimated. Therefore, collaborative effort between governments of developing countries, policymakers, researchers, and general public is imperative to reduce incidence of foodborne diseases. Use of rapid methods for detection of foodborne pathogens is required in developing countries. Human capacity development in state-of-art technologies and foodborne pathogen detection methods among researchers in developing countries in collaboration with researchers in developed countries is also encouraged for the prevention of transmission and awareness of foodborne diseases.

*Olumide A. Odeyemi* Springforth Scientific Resource Centre Ile-Ife, Osun State, Nigeria Email: oluodeyemi@gmail.com
